# Supplementary material for: C-type natriuretic peptide/cGMP/FoxO3 signaling attenuates hyperproliferation of pericytes from patients with pulmonary arterial hypertension
Source: Commun Biol. 2024 Jun 6;7:693. doi: 10.1038/s42003-024-06375-3 (PMC11156916; doi:10.1038/s42003-024-06375-3)

Supplementary information

Supplementary Table 1: List of siRNAs

Name	Company	Sequence
FoxO3 siRNA	Thermo Scientific (VHS41092)	AUUGACCAAACUUCCCUGGUUAGGC
Control siRNA	Qiagen (10277281)	Proprietary sequence

Supplementary Table 2: List of Antibodies

**Name**	**Company**	**Concentration**
Phospho FoxO1/FoxO3 (Thr24/32)	Cell Signaling (#2599)	1: 1000
FoxO3	Cell Signaling (#12829)	1: 1000
FoxO3	Cell Signaling (#2497)	1: 200 (Immunocytochemistry)
Phospho VASP (Ser239)	Cell Signaling (#3114)	1: 1000
VASP	Cell Signaling (#3112)	1: 1000
Phospho AKT (Ser473)	Cell Signaling (#4060)	1: 1000
AKT	Cell Signaling (#9272)	1: 2000
Phospho ERK 1/2 (Thr202/Tyr204))	Cell Signaling (#9101)	1: 1000
ERK	Cell Signaling (#9102S)	1: 2000
Phospho PTEN (Ser380/Thr382/383)	Cell Signaling (#9554)	1:1000
PTEN	Cell Signaling (#9559)	1:1000
cGKI	Cell Signaling (#3248)	1:1000
Cyclin D1	Santa Cruz (sc-8396)	1:1000
GAPDH	Cell Signaling (#2118)	1:10000
Na^+-K^+ ATPase	Abcam (ab76020)	1:2000
GC-B	Used in previous publication [12]	1: 1000
αSMA	Sigma (A5228)	1:5000
PCNA	Santa Cruz (sc-56)	1:1000

Supplementary 3: List of Primers

Gene name	Forward primer	Reverse primer
B2M (Human)	CACCCCCACTGAAAAAGATGAG	CCTCCATGATGCTTACATG
CNP (human)	CGGCCTGGGATGTTAGTG	AAAGATGACCTCAGCACAACG
GC-B (human)	TGTGTATATCTGCGGCCCTC	CGGGCTCTTATCAGCAGACG
B2M (rat)	TCGGTGACCGTGATCTTTCT	GTTGGGCTTCCCATTCTCCG
CNP (rat)	GGATGTTAGTGCAGCGACCC	ATTGCGTTGGAGGTGTTTCC
GC-B (rat)	GTTTGGTGTTTCCAGCTTCCT	TCATGAGCGAGCCGTAACT
GAPDH (mouse)	ATGGTGAAGGTCGGTGTGA	AATCTCCACTTTGCCACTGC
CNP (mouse)	AGCGGTCTGGGATGTTAGTG	CGTTGGAGGTGTTTCCAGAT
GC-B (mouse)	TGTTTGGTGTTTCCAGTTTCC	AGTTCTTCCCAGCGAATGC

Supplementary Figure 1: PDGFR-β and TGF-β RII protein expression is upregulated in PAH pericytes.

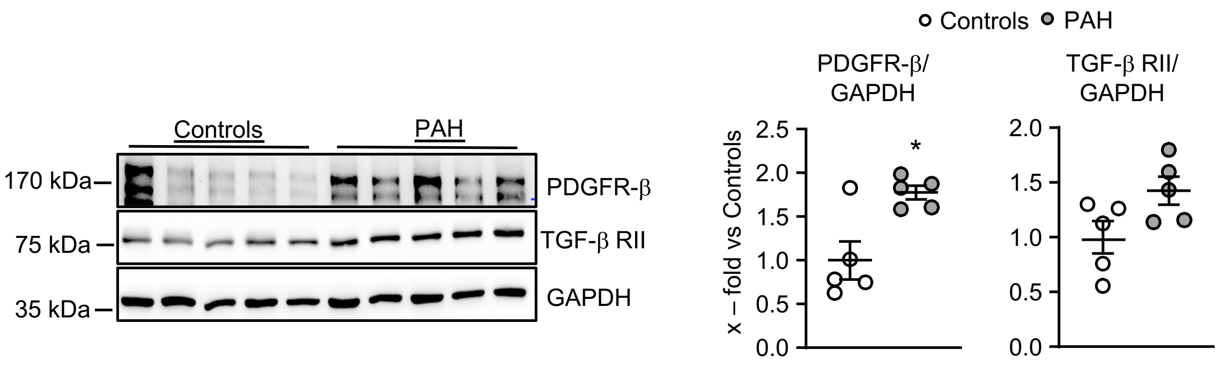

Suppl. Figure 1. Lung pericytes isolated from patients with PAH display higher expression of PDGFR-β and TGF-β RII in comparison to pericytes from control individuals as analysed by immunoblotting (n = 5 biological replicates from controls and PAH patients (unpaired 2-tailed Student's t test). *$p < 0.05$ vs. controls.

Supplementary Figure 2: Rp-8-Br-PET-cGMPS inhibits cGKI activity but does not prevent the inhibitory effect of CNP on PDGF-BB mediated ERK phosphorylation.

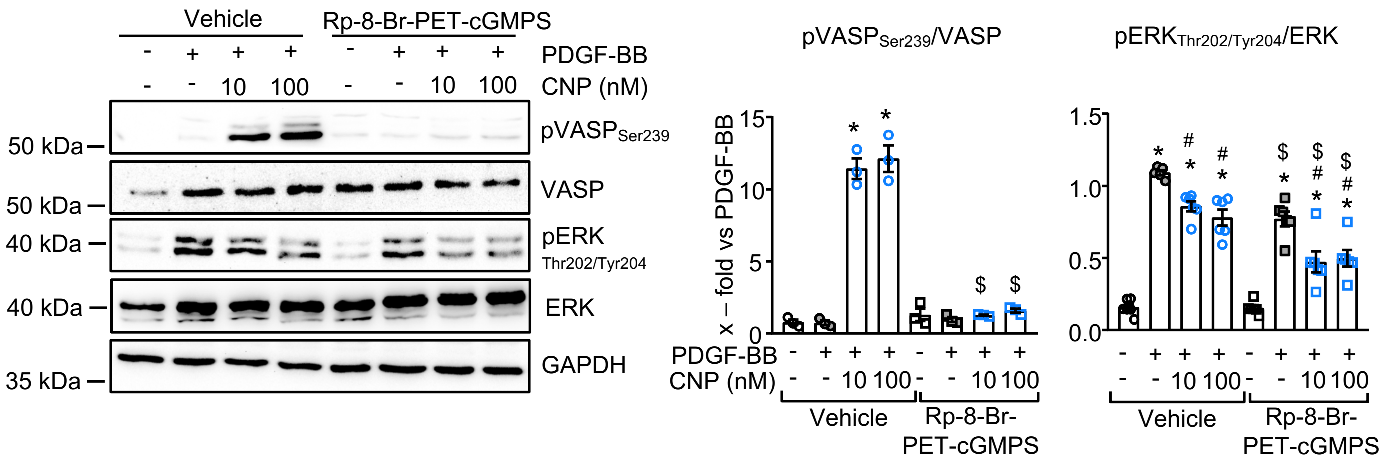

Suppl. Figure 2. The cGKI inhibitor Rp-8-Br-PET-cGMPS (100 µM) prevented the stimulatory effect of CNP on phosphorylation of VASP (Ser_{239}) but did not attenuate CNP effects on PDGF-BB (30 ng/ml) induced phosphorylation of ERK (Thr_{202}/Tyr_{204})**. (**n = 3-6 from 3 biological replicates, 2-way ANOVA). **p*<0.05 vs PBS, $^{\#}p<0.05$ vs PDGF-BB, $^{\$}$p<0.05 vs corresponding vehicle-treated group.

Supplementary Figure 3. cGKI activation mimics the inhibitory effects of CNP on PDGF-BB-induced proliferative signaling.

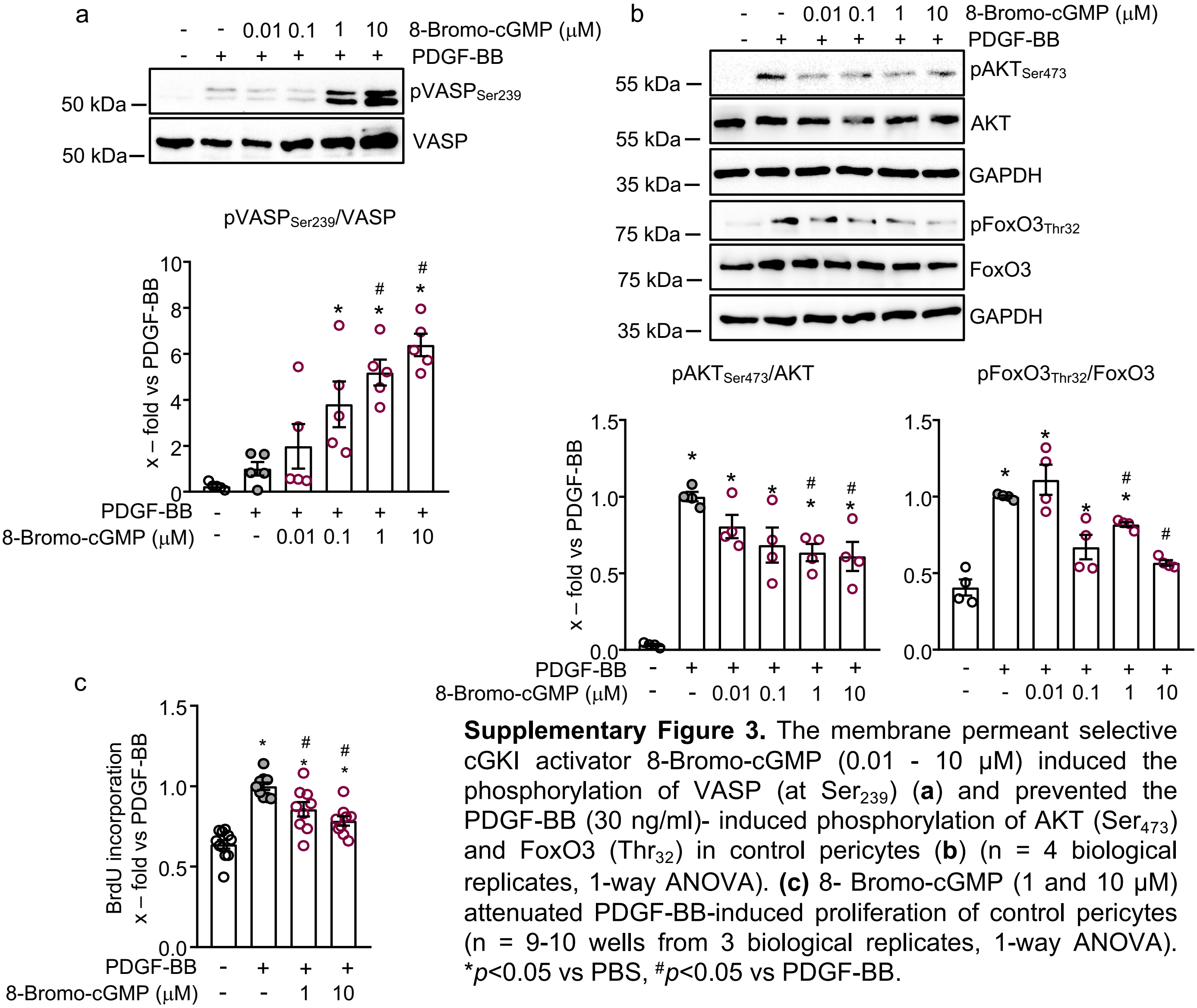

Supplementary Figure 3. The membrane permeant selective cGKI activator 8-Bromo-cGMP (0.01 - 10 μM) induced the phosphorylation of VASP (at Ser_{239}) (**a**) and prevented the PDGF-BB (30 ng/ml)- induced phosphorylation of AKT (Ser_{473}) and FoxO3 (Thr_{32}) in control pericytes (**b**) (n = 4 biological replicates, 1-way ANOVA). **(c)** 8- Bromo-cGMP (1 and 10 μM) attenuated PDGF-BB-induced proliferation of control pericytes (n = 9-10 wells from 3 biological replicates, 1-way ANOVA). *p<0.05 vs PBS, $^{\#}p$<0.05 vs PDGF-BB.

Supplementary Figure 4: Uncropped blots

Figure 1: Uncropped western blots

Figure 1c

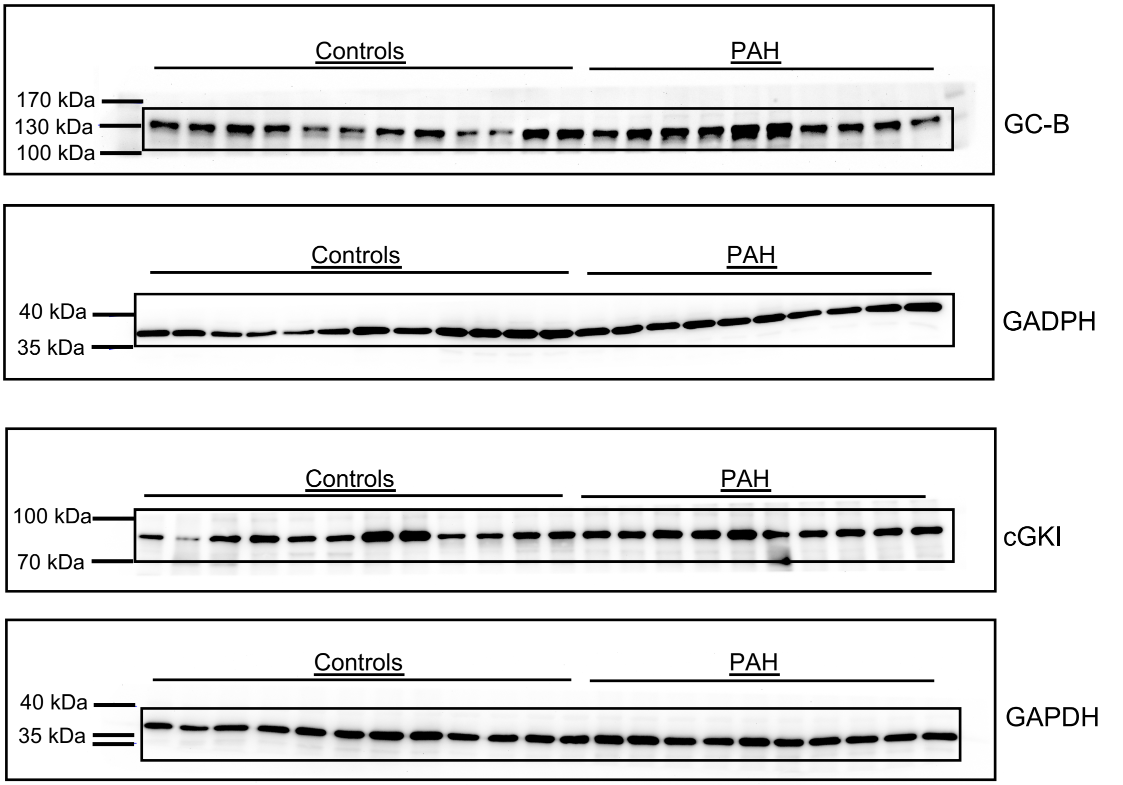

Figure 1d

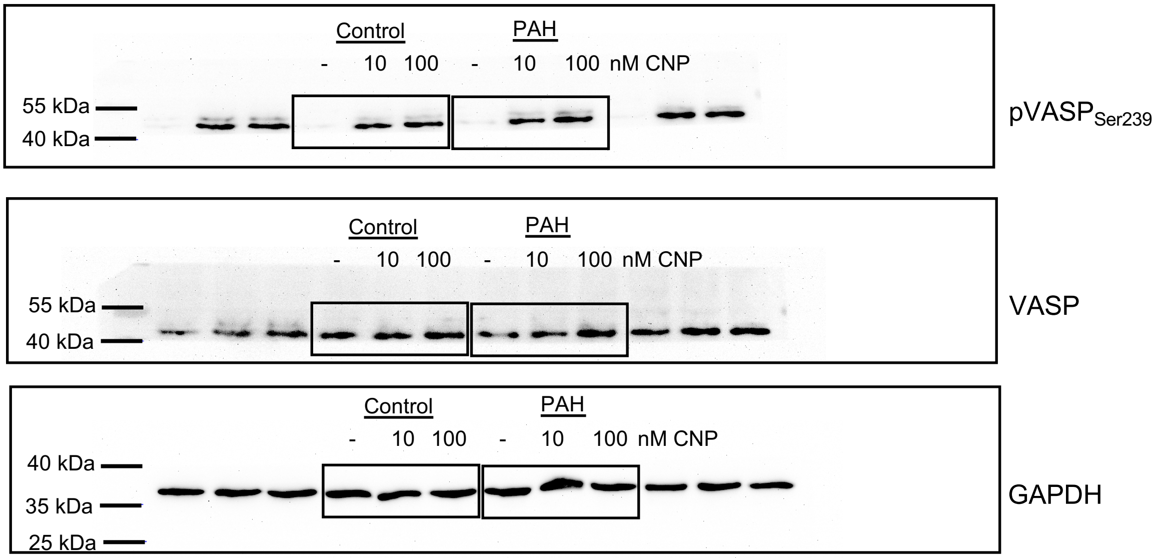

Figure 2: Uncropped western blots

Figure 2e

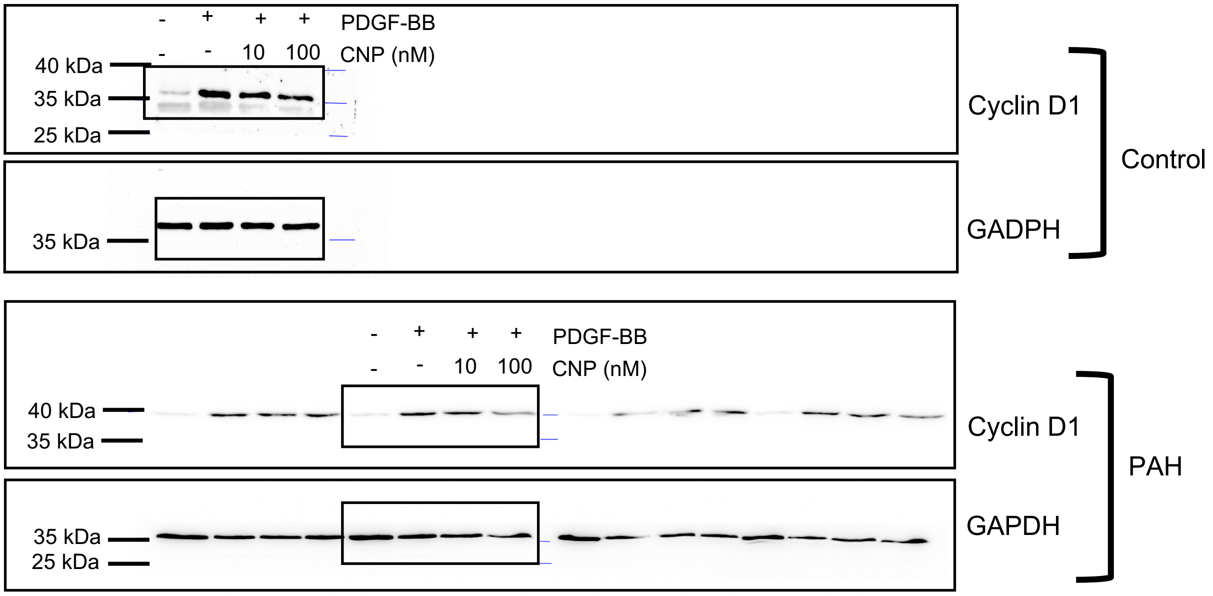

Figure 2f

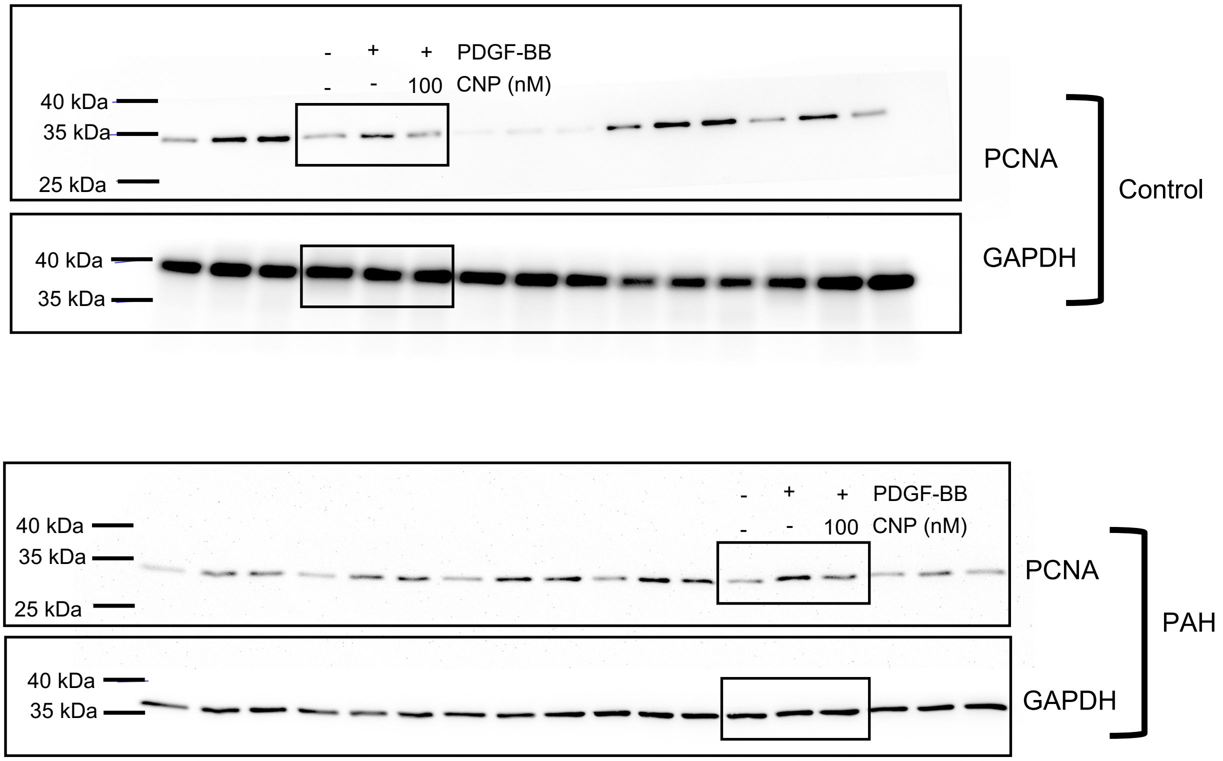

Figure 3: Uncropped western blots

Figure 3b

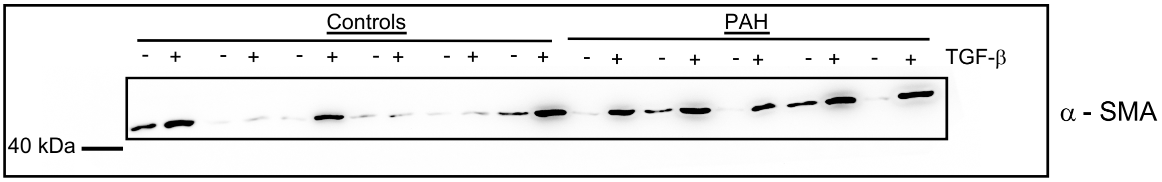

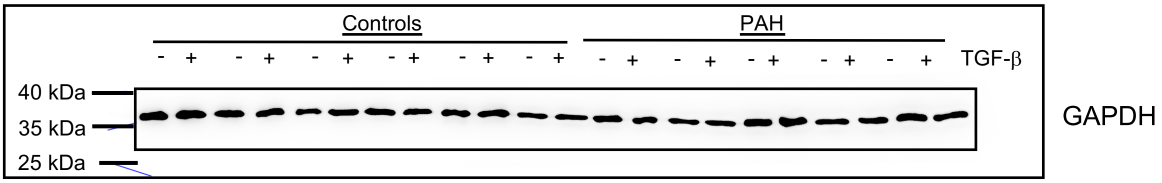

Figure 3c

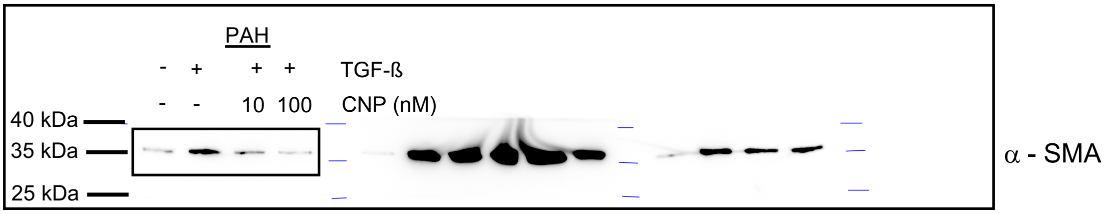

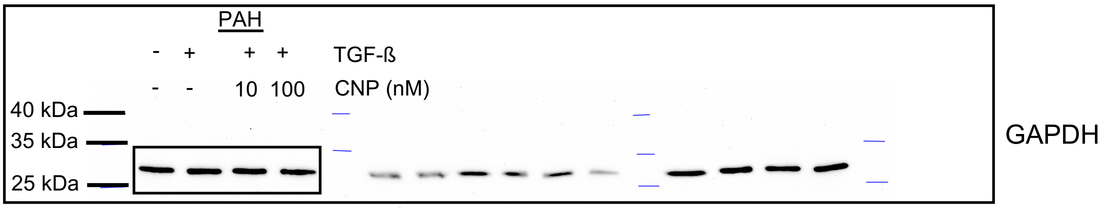

Figure 4: Uncropped western blots

Figure 4a

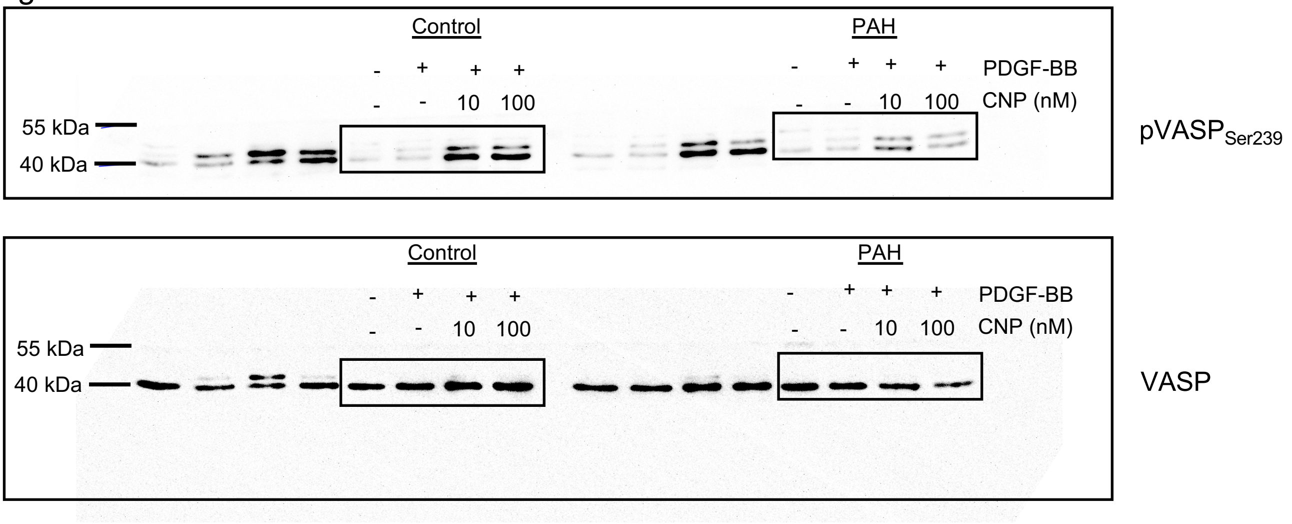

Figure 4b

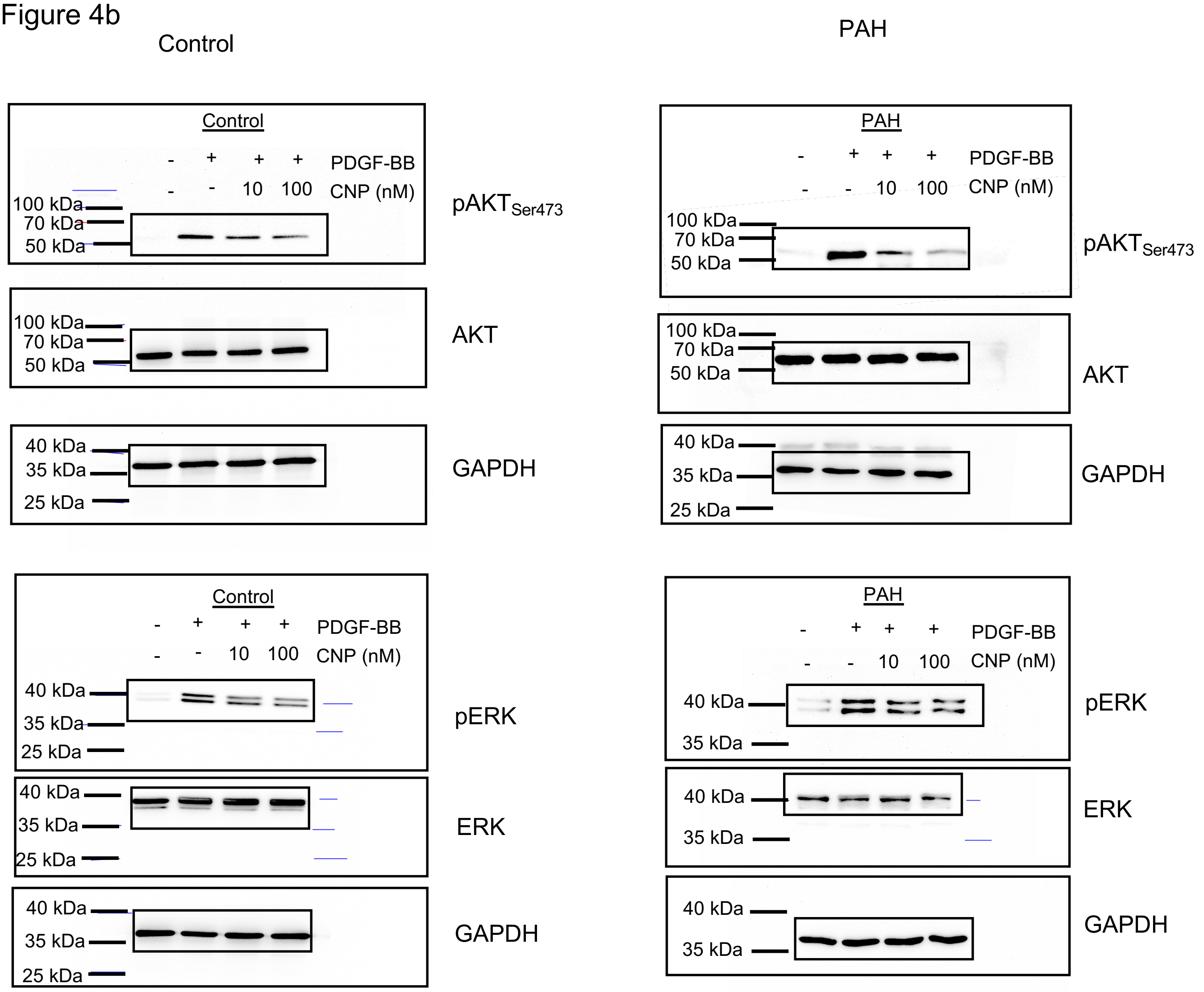

Figure 5: Uncropped western blots

Figure 5a

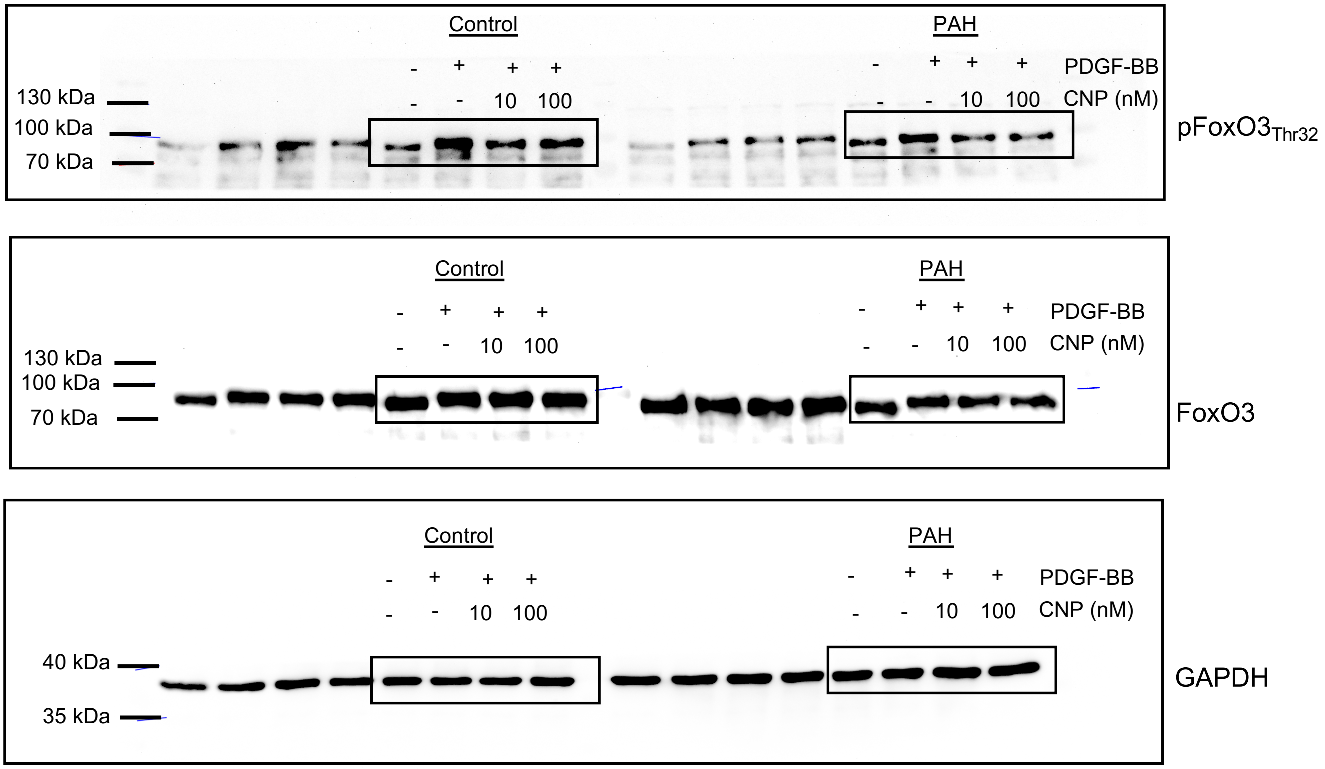

Figure 5c

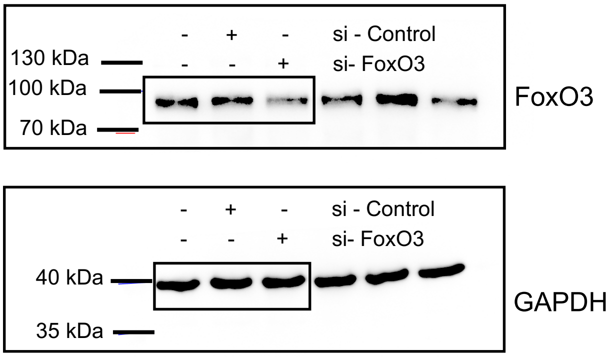

Figure 6: Uncropped western blots

Figure 6a

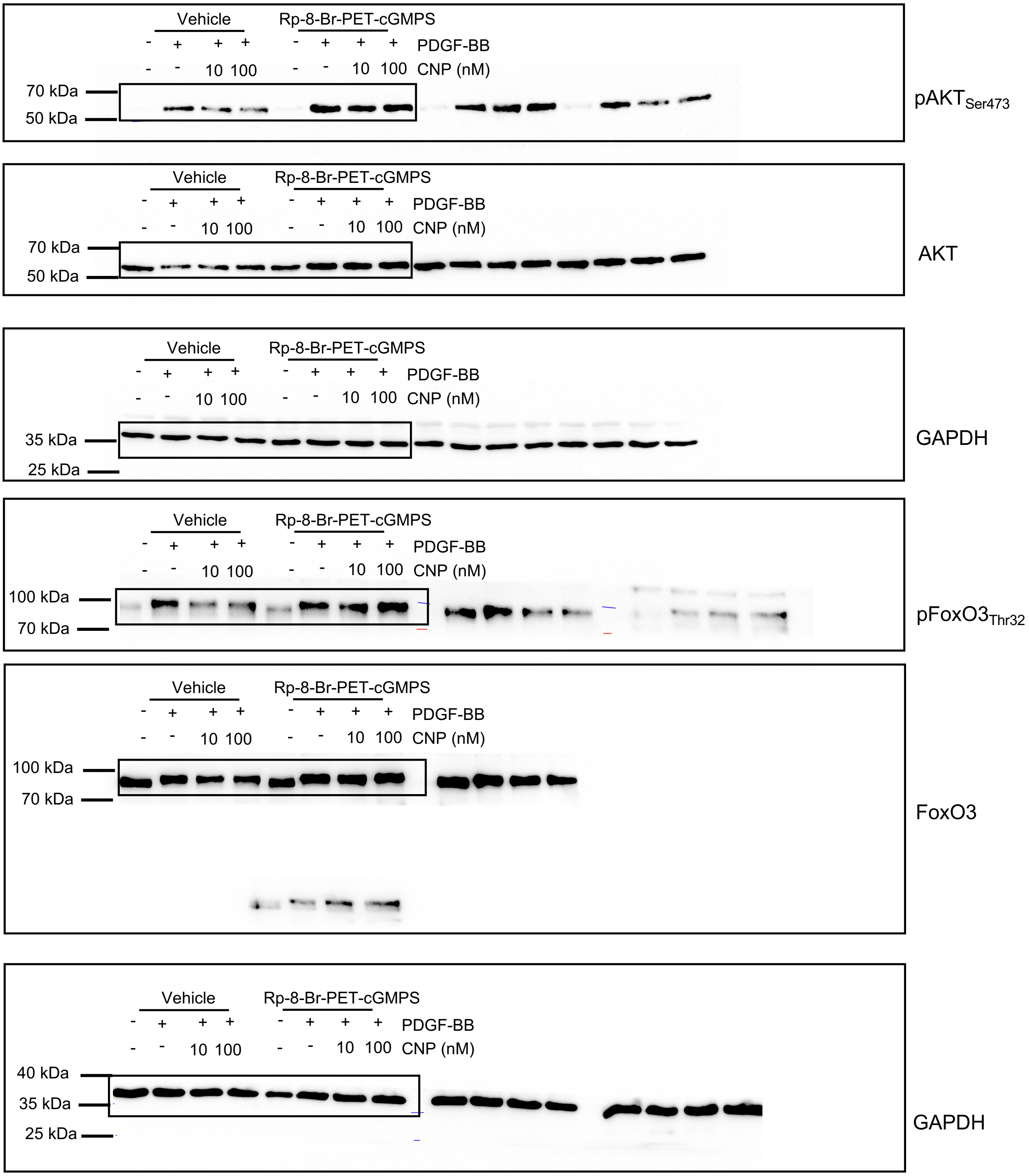

Figure 6c

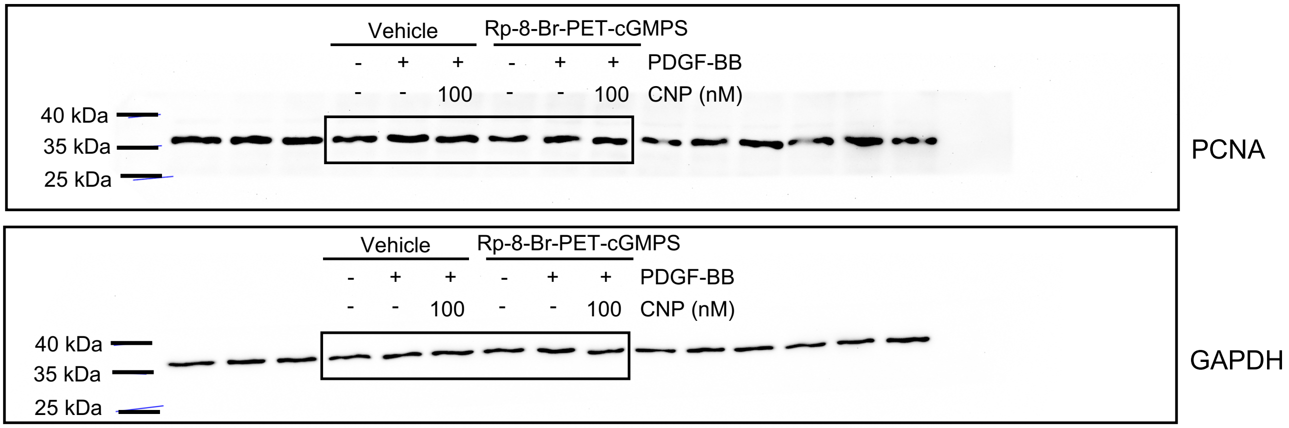
Vehicle
Rp-8-Br-PET-cGMPS
- + + - + + PDGF-BB
- - 100 - - 100 CNP (nM)
40 kDa
35 kDa
25 kDa
PCNA
Vehicle
Rp-8-Br-PET-cGMPS
- + + - + + PDGF-BB
- - 100 - - 100 CNP (nM)
40 kDa
35 kDa
25 kDa
GAPDH

Figure 7: Uncropped western blots

Figure 7b

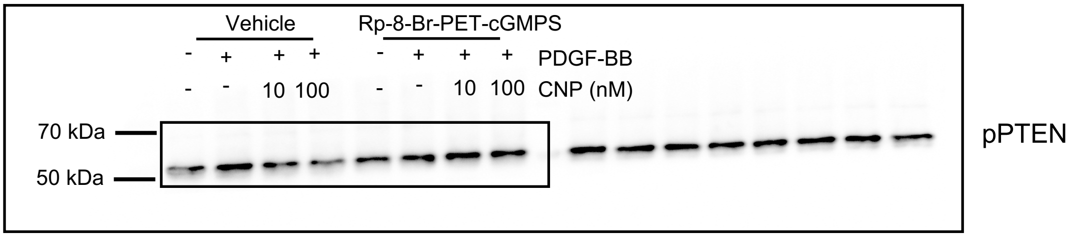

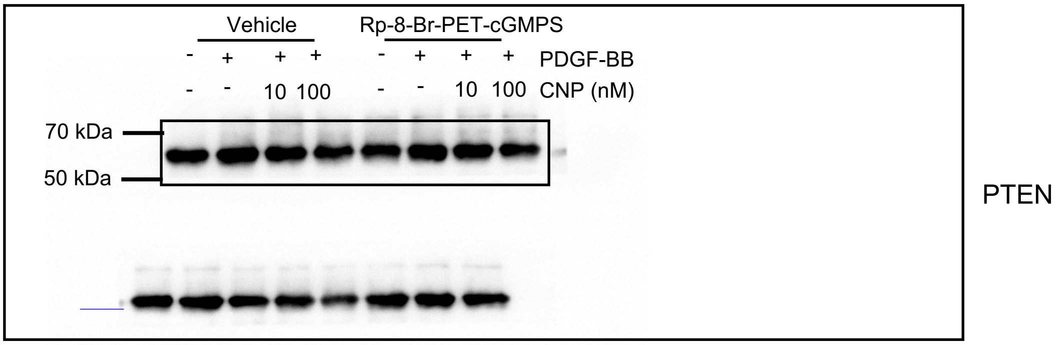

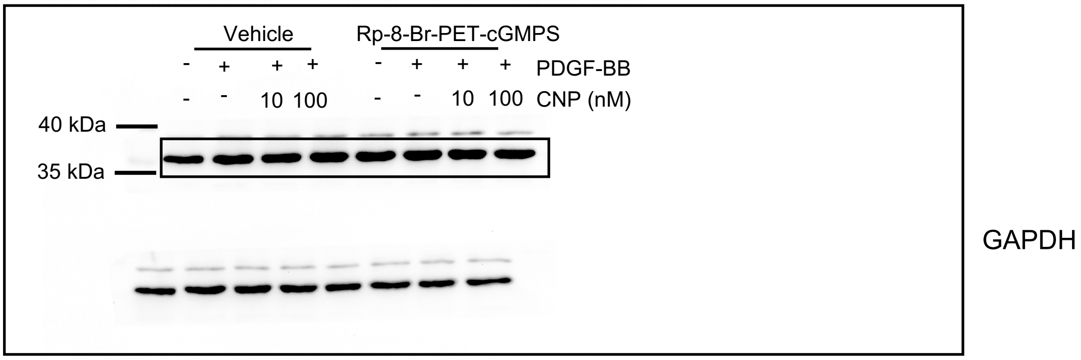

Figure 7c

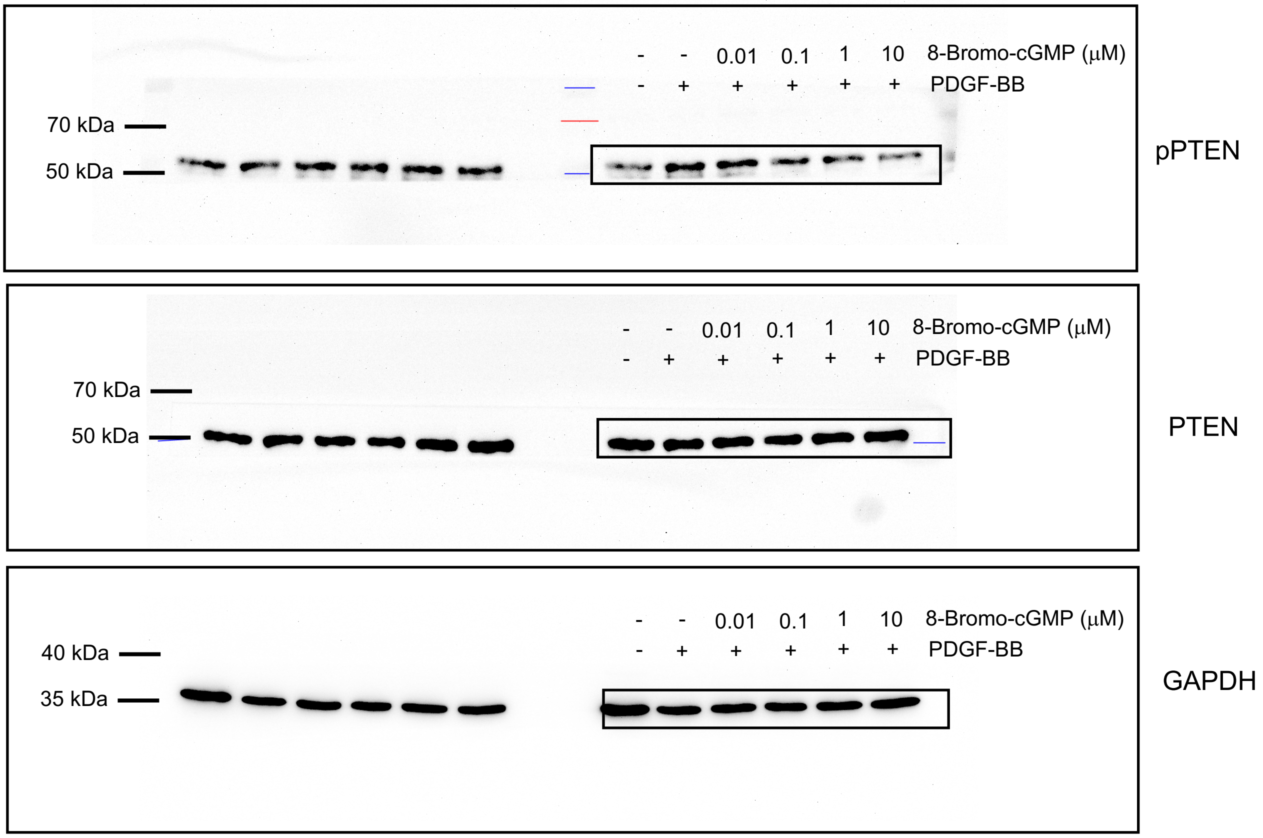
- - 0.01 0.1 1 10 8-Bromo-cGMP (μM)
- + + + + + PDGF-BB
70 kDa
50 kDa
pPTEN
- - 0.01 0.1 1 10 8-Bromo-cGMP (μM)
- + + + + + PDGF-BB
70 kDa
50 kDa
PTEN
- - 0.01 0.1 1 10 8-Bromo-cGMP (μM)
- + + + + + PDGF-BB
40 kDa
35 kDa
GAPDH

Suppl. Figure 1: Uncropped western blots

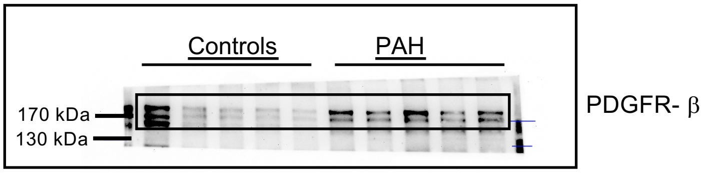

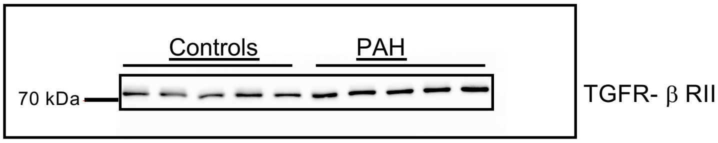

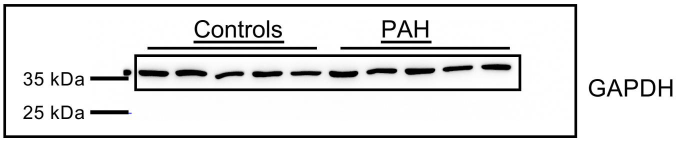

Suppl. Figure 2: Uncropped western blots

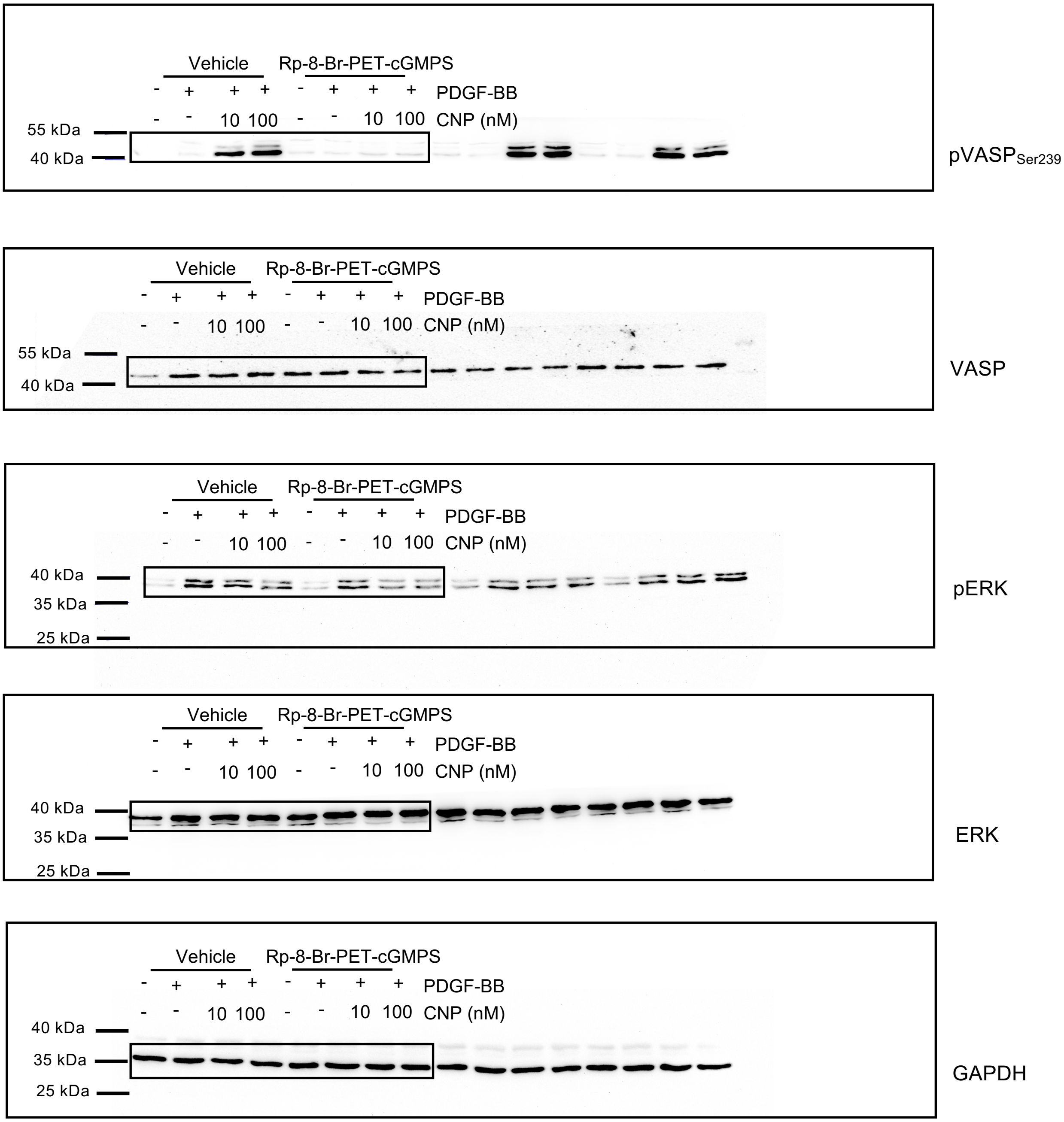

Suppl. Figure 3: Uncropped western blots

Suppl. Figure 3a

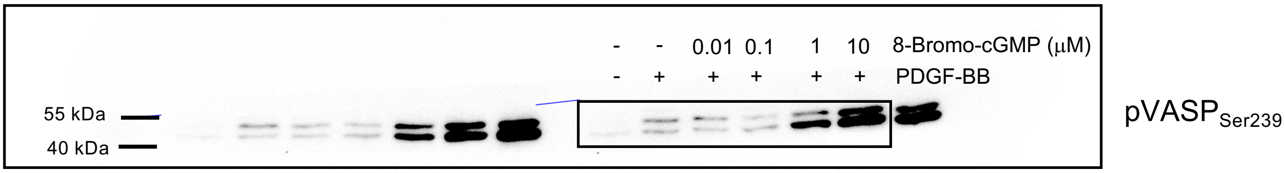

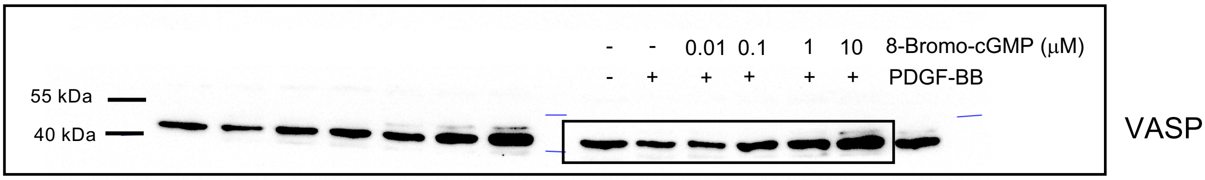

Suppl. Figure 3b

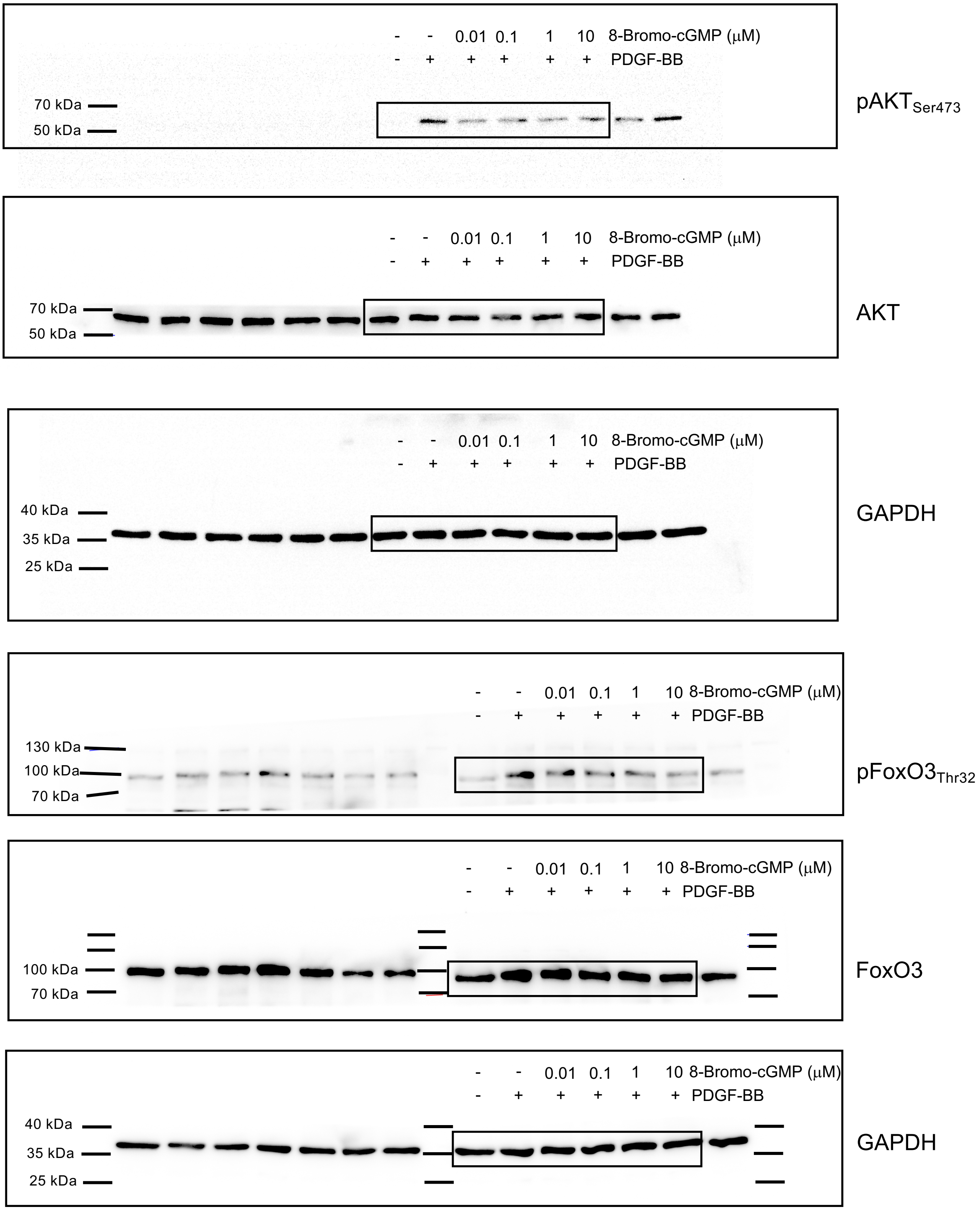

Supplement: Supplementary file 2 — Supplementary Information [file 42003_2024_6375_MOESM2_ESM.pdf]
